# Axial distribution of myosin binding protein-C is unaffected by mutations in human cardiac and skeletal muscle

**DOI:** 10.1007/s10974-012-9286-9

**Published:** 2012-03-14

**Authors:** Anupama Vydyanath, Christina A. Gurnett, Steve Marston, Pradeep K. Luther

**Affiliations:** 1 Section of Molecular Medicine, Faculty of Medicine, National Heart and Lung Institute, Imperial College London, London, SW7 2AZ UK; 2 Division of Paediatric Neurology, Department of Neurology, Washington University School of Medicine, St Louis, MO 63110 USA; 3 Myocardial Function, National Heart and Lung Institute, Imperial College London, London, SW7 2AZ UK

**Keywords:** Hypertrophic cardiomyopathy, Distal arthrogryposis type 1, C-protein, MyBP-C, Myosin binding protein-C mutations, Thick filament

## Abstract

Myosin binding protein-C (MyBP-C), a major thick filament associated sarcomeric protein, plays an important functional and structural role in regulating sarcomere assembly and crossbridge formation. Missing or aberrant MyBP-C proteins (both cardiac and skeletal) have been shown to cause both cardiac and skeletal myopathies, thereby emphasising its importance for the normal functioning of the sarcomere. Mutations in cardiac MyBP-C are a major cause of hypertrophic cardiomyopathy (HCM), while mutations in skeletal MyBP-C have been implicated in a disease of skeletal muscle—distal arthrogryposis type 1 (DA-1). Here we report the first detailed electron microscopy studies on human cardiac and skeletal tissues carrying MyBP-C gene mutations, using samples obtained from HCM and DA-1 patients. We have used established image averaging methods to identify and study the axial distribution of MyBP-C on the thick filament by averaging profile plots of the A-band of the sarcomere from electron micrographs of human cardiac and skeletal myopathy specimens. Due to the difficulty of obtaining normal human tissue, we compared the distribution to the A-band structure in normal frog skeletal, rat cardiac muscle and in cardiac muscle of MyBP-C-deficient mice. Very similar overall profile averages were obtained from the C-zones in cardiac HCM samples and skeletal DA-1 samples with MyBP-C gene mutations, suggesting that mutations in MyBP-C do not alter its mean axial distribution along the thick filament.

## Introduction

**Fig. 1:**
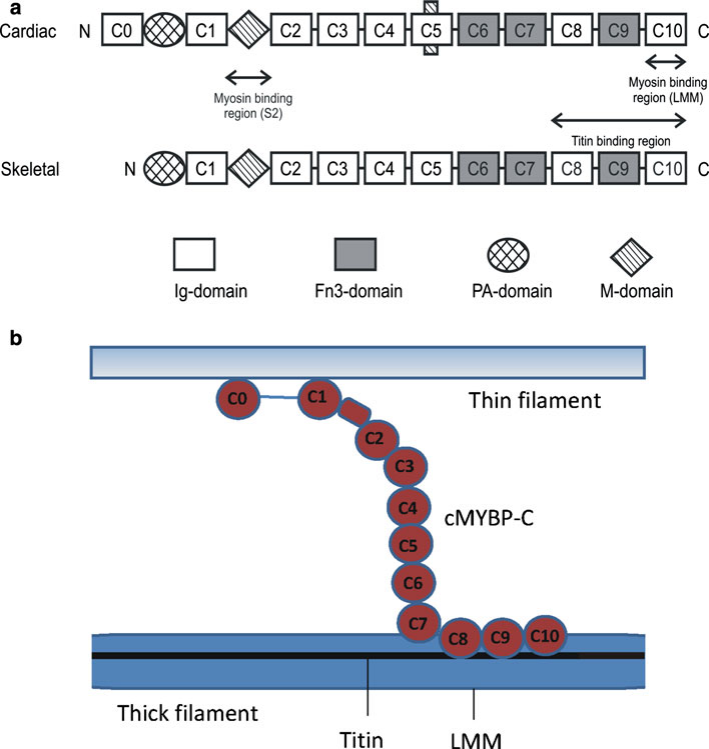
Organisation of MyBP-C. **a** Schematic showing the domain architecture of cardiac and skeletal MyBP-C. The protein is composed of repeats of IgI-like and FnIII-like domains. PA-domain refers to the proline–alanine rich linker region while M-domain refers to the 105 residue domain between C1 and C2 also called MyBP-C motif. **b** Schematic arrangement of cardiac MyBP-C (cMyBP-C) with respect to the components of the sarcomere. Here the C-terminal of cMyBP-C is arranged parallel to the thick filament backbone (Squire et al. 2003), while the N-terminal is shown interacting with a thin filament

Myosin binding protein-C (MyBP-C) is a 140 kDa sarcomeric protein that binds to the thick filament in vertebrate striated muscle and can be visualised as 7–9 stripes of separation 43 nm in the C-zone of the sarcomere. It was discovered in skeletal muscle nearly 40 years ago by Offer et al. (1973) as a contaminant in the preparation of purified myosin. There are three isoforms, slow skeletal, fast skeletal and cardiac, encoded by the genes *MYBPC1*, *MYBPC2* and *MYBPC3* and located on chromosome 12, 19 and 11 respectively (for reviews see Flashman et al. 2004; Harris et al. 2011; Schlossarek et al. 2011). All three isoforms share a conserved domain architecture, composed of seven immunoglobulin (IgI) domains and three fibronectin type III (FnIII) domains depicted as C1–C10 (Fig. 1a), with a 105 residue domain between C1 and C2 called the M-domain or MyBP-C motif and a proline- and alanine-rich region near the N-terminus (Gautel et al. 1995; Okagaki et al. 1993). The cardiac isoform differs from the skeletal isoform in three major ways: it has an additional Ig domain, C0, at the N-terminus; it has 3 phosphorylation sites in the M-domain and the domain C5 has a proline-rich 25 residue insertion (Gautel et al. 1995). The homology of the amino acids in all three isoforms is high, with 39.6 % sequence identity in the human isoforms.

There is evidence that suggests that MyBP-C interacts with thick filaments, thin filaments and titin. It binds to light meromyosin portion of the myosin rod (LMM) and titin via its C-terminal domains C7–C10 (Freiburg and Gautel 1996). In vitro experiments show that MyBP-C can modulate actin–myosin interaction by binding to actin filaments (Moos et al. 1978; Kulikovskaya et al. 2003; Shaffer et al. 2009). This has been visualised in a recent thick filament reconstruction which showed clear MyBP-C density perpendicular to the filament, extending beyond the myosin heads, and making contact with actin filaments (Luther et al. 2011). Furthermore, a weak interaction has also been observed in vitro between MyBP-C and myosin S2, and this interaction is mediated through domains C1–C2 (Starr and Offer 1978; Gruen and Gautel 1999). A schematic arrangement of the cardiac isoform of MyBP-C with respect to the filament systems is shown in Fig. 1b. The precise arrangement of the C-terminus of MyBP-C in the sarcomere with respect to myosin remains unresolved, since there is evidence that suggests both a trimeric collar arrangement (Moolman-Smook et al. 2000) and an axial arrangement (Squire et al. 2003; Zoghbi et al. 2008). The interaction between MyBP-C, myosin, and titin is believed to be responsible for maintaining the ordered arrangement of the sarcomere (Freiburg and Gautel 1996). Furthermore phosphorylation of the cardiac isoform modulates contractility and also regulates cross-bridge kinetics (Barefield and Sadayappan 2010).

The importance of MyBP-C for the normal functioning of the sarcomere is further emphasised by the occurrence of cardiac and skeletal myopathies due to missing or aberrant MyBP-C proteins. Mutations in *MYBPC3* encoding cardiac MyBP-C (cMyBP-C) are a major cause of hypertrophic cardiomyopathy (HCM), a disease that is estimated to affect 1 in 500. It is characterised by a thickening of the left ventricular wall, significant myofibrillar disarray and interstitial fibrosis. Mutations in *MYBPC1* encoding slow-skeletal MyBP-C (ssMyBP-C) have recently been implicated in a disease of skeletal muscle—distal arthrogryposis type 1 (DA-1)—characterised by congenital contracture of distal limbs. So far two DA-1 causing *MYBPC1* mutations have been identified, while 197 HCM causing *MYBPC3* mutations have been reported (Alcalai et al. 2008; Gurnett et al. 2010; for reviews see Harris et al. 2011 and Schlossarek et al. 2011). Both diseases are transmitted as autosomal-dominant traits, but studies by Gurnett et al. (2010), indicate that the *MYBPC1* and *MYBPC3* mutations may adopt different disease causing mechanisms.

Although research into *MYBPC1* mutations is relatively limited, the mutations in *MYBPC3* (cardiac isoform) have been more extensively researched for the last 15 years. Remarkable headway has been made in understanding the genetic mechanisms and functional consequence of the *MYBPC3* mutations in HCM. Work has been carried out by Luther et al. (2008) using electron microscopy to visualise healthy, small animal and cMyBP-C knockout mouse sarcomeres (cardiac and skeletal). However the structural consequence of these mutations to the thick filament and sarcomere architecture in the diseased human tissues is relatively unknown. Investigation into the ultrastructural organisation of the sarcomere can help better understand the functional shortcoming observed in these myopathies. In this study electron microscopy and A-band analysis of human cardiac and skeletal tissue from patients with *MYBPC1* or *MYBPC3* mutations has been carried out. Specifically we investigated the effect of the mutations on the presence and axial distribution of MyBP-C into the C-zone of the sarcomere.

## Experimental procedures

### Sample preparation

#### Skeletal

Muscle biopsy samples were conventionally processed and embedded in Araldite epoxy resin. Semi-thick 0.5 μm sections and thin 100 nm sections were cut with a Reichert Ultracut E ultramicrotome. Thin sections picked on copper grids were stained with 2 % uranyl acetate and Reynolds lead citrate. The biopsy sample details are listed in Table 1. In this manuscript, the two skeletal mutations will be referred to as ss-W236R and ss-Y856H, respectively. The frog skeletal samples were used as described in Luther et al. (2008).

**Table 1:** Details of the myectomy and biopsy samples used in this study and the MyBP-C gene mutations identified

Isoform	Mutation	Sample code and detail	Domain affected	Diagnosis	Reference
cMyBP-C	c.1624G>C	MH1 fresh tissue	E542Q amino acid substitution in the C3 domain or predicted truncation at C3 domain due to exon skipping	HCM	Marston et al. (2011)
cMyBP-C	InsG2374	M9 frozen sample, homogenised.	Truncation at the C5 domain	HCM	Marston et al. (2009)
cMyBP-C	c.472G>A	M4 frozen sample, homogenised.	V158M Polymorphism	HCM	Marston et al. (2009)
cMyBP-C	None	M24 fresh tissue, cryosections prepared for em	Myectomy control	HCM	Marston et al. (2009)
ssMyBP-C	c.706T>C	ss-W236R fresh tissue	W236R amino acid substitution in the MyBP-C motif	DA-1	Gurnett et al. (2010)
ssMyBP-C	c.2566T>C	ss-Y856H fresh tissue	Y856H amino acid substitution in the C8 domain	DA-1	Gurnett et al. (2010)

#### Cardiac

Myectomy samples were obtained either as freshly excised tissue or snap frozen in liquid nitrogen. Freshly excised cardiac tissue obtained directly after the myectomy procedure were incubated straightaway for 30 min in oxygenated Krebs solution (94.5 mM sodium chloride, 5 mM potassium chloride, 25 mM sodium hydrogen carbonate, 1 mM disodium hydrogen phosphate, 1 mM magnesium sulphate·7H_2_O, 10 mM sodium acetate, 10 mM glucose and 1 mM calcium chloride, pH 7.4) with 30 mM 2,3-butanedione monoxime (BDM) and then fixed in 3 % gultaraldehyde in Krebs solution for 1 h, followed by secondary fixation in 1 % osmium tetroxide for 30 min. Dehydration using increasing concentrations of acetone (50, 70, 80, 90, 95, 100 % and finally dry acetone) was then carried out. The sample was subsequently embedded into the epoxy resin Araldite CY 212. Alternatively, after fixation in 3 % glutaraldehyde, the tissue was prepared for cryosectioning following the protocol described by Luther et al. (2008).

Some of the myectomy samples were snap frozen in liquid nitrogen. These samples were thawed and homogenised in cold relaxing solution (100 mM potassium chloride, 20 mM imidazole, 2 mM magnesium chloride, 2 mM K_2_EGTA and 4 mM ATP) with 30 mM BDM (10 ml) using a Polytron homogeniser and the resultant suspension spun at 1,200 rpm for 1 min. The resulting pellet was incubated in a skinning solution (relaxing solution with 0.3 ml of 1 % Triton X) for 7 min (with frequent rolling) before being spun again (1,200 rpm/1 min). The sample was then spun twice more with fresh relaxing solution (10 ml at room temperature) and to the resultant sample cold relaxing solution (1 ml) was added and left for 30 min over ice. The sample was then applied onto Thermonox cover-slips with a Cytospin 3 and chemically fixed with 3 % glutaraldehyde in relaxing solution for 15 min. Secondary fixation, dehydration and embedding steps were performed as described in the preceding paragraph.

Semi-thick 0.5 μm and thin 100 nm sections from araldite blocks were cut with a Reichert Ultracut E ultramicrotome. Thin sections were picked on copper grids and stained with 2 % uranyl acetate and Reynolds lead citrate. Cryosections of frozen samples ~100 nm thick were cut with an RMC MT7 ultramicrotome fitted with a CR20 cryoattachment, transferred to formvar/carbon-coated nickel grids, and negative-stained with 2 % uranyl acetate. The myectomy sample details are listed in Table 1. In this manuscript, the cardiac mutation will be referred by their sample codes as shown in Table 1. The preparation of rat cardiac and cMyBP-ko mouse samples has been described in detail in Luther et al. (2008).

### Histology studies

The 0.5 μm semi-thick sections were stained with Paragon stain (0.73 % toluidine blue, 0.27 % Basic Fuchsin in 30 % ethanol) by combining the stain with saturated borax solution in a 4:1 ratio on the slide. The slides were examined using a Nikon inverted microscope using 20× objective and 100× oil immersion objective and photographed with an Olympus E330 camera.

### Electron microscopy

A JEOL 1200 EX electron microscope operated at 100 kV was used to examine the grids. Images for the frog skeletal muscle, rat cardiac muscle and three of the cardiac myectomy tissues were recorded on electron microscope film (Kodak SO-163) at 20,000× magnification and the films scanned with a Nikon Coolscan 8000ED 4,000 dpi scanner and binned four times to give a pixel resolution of about 1.3 nm. Images for all the other samples were recorded with a Tietz Fastscan CCD camera and Tietz EMMenu 4.0 software was used to view and record images at a range of magnifications (2,500×–15,000×).

### Method for averaging profile plots

The images were analysed as described by Luther et al. (2008), using custom software written by C. Knupp and ImageJ software. Briefly, longitudinal regions were first rotated to make the cross bridge striations vertical, then suitable half sarcomeres were boxed, band-pass filtered and the profile plot data collected. The 1-D profile plots obtained were averaged by cross-correlation to produce an average axial density plot.

## Results

Human skeletal muscle was available from two patients with slow skeletal *MYBPC1* mutations obtained from abductor hallucis muscle open biopsies (Gurnett et al. 2010). Both mutations occur in highly conserved regions. The first mutation, W236R, occurs in the MyBP-C motif, while the second mutation, Y856H, occurs in the C8 domain.

Human cardiac samples used in this study were obtained from inter-ventricular septum of HCM patients undergoing surgical myectomy to relieve hypertrophic obstructive cardiomyopathy. Four HCM myectomy samples were analysed in this study (details in Table 1). Three of the samples were identified with *MYBPC3* mutations (MH1, M9, and M4). MH1 was identified with G>C transversion on the last nucleotide of exon 17 (c.1624G>C). This mutation is predicted to produce either a mutant full length protein with a missense E542Q mutation in the C3 domain or lead to the skipping of exon 17 resulting in a premature termination of the translation in the middle of C3 domain of the molecule (Carrier et al. 1997). Direct measurements showed substantial amounts of nonsense mRNA due to skipping of exon 17 and there was a 28 % deficiency of MyBP-C in this muscle (Marston et al. 2011). M9 was identified as InsG2374, giving rise to polypeptide with a premature termination at the C5 domain and is 90 kDa in size. This peptide is not present in the muscle and haploinsufficiency of 19 % was reported (Marston et al. 2009). M4 contains a V158M mutation which was attributed to a gene polymorphism that is unlikely to be disease causing (Marston et al. 2009). No gene mutation was identified in M24 and was therefore used as myectomy control in this study. Details of all the samples used in this study are listed in Table 1.

### Histological analysis of the human samples

**Fig. 2:**
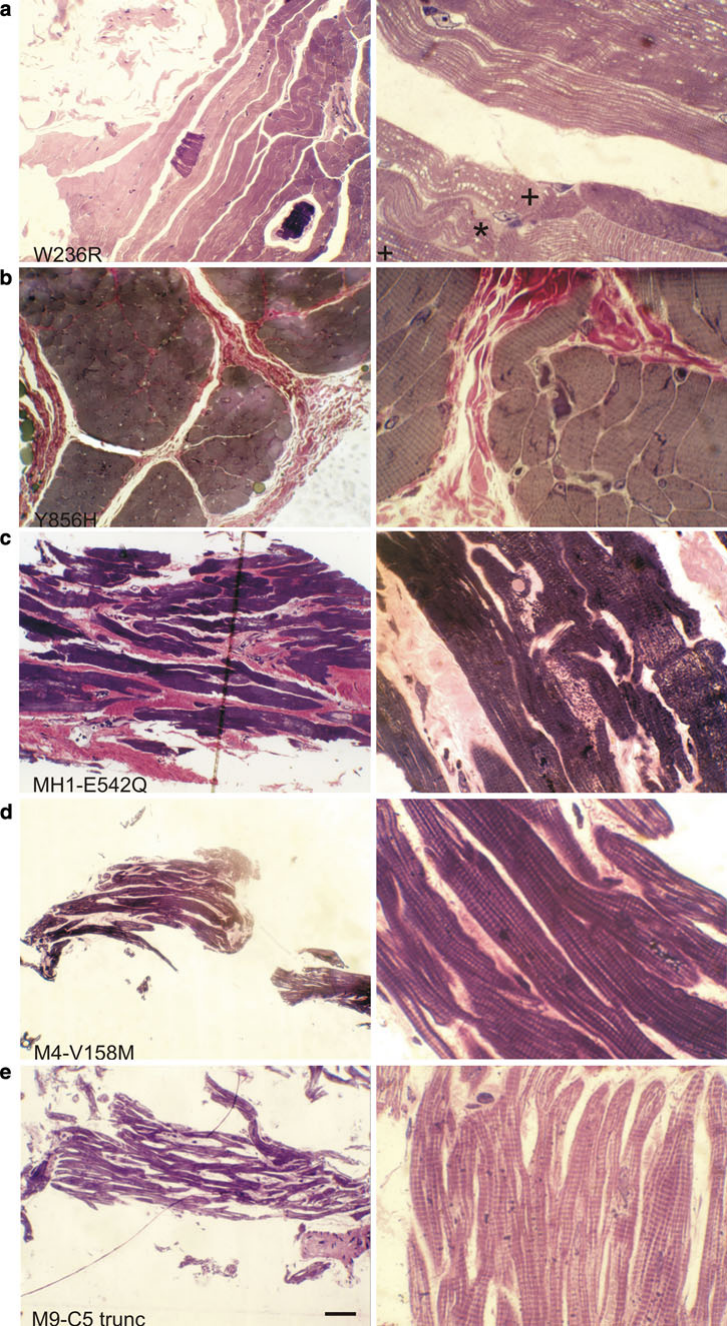
Light microscopy analysis of muscle biopsy and cardiac myectomy samples taken at ×20 (LHS panels) and ×100 (RHS panels). Paragon staining of semi thin sections of **a** ss-W236R, **b** ss-Y856H, **c** MH1, **d** M4 and **e** M9 is shown; the stain highlights cellular components in* dark blue* and connective tissue* pink*. Sarcomeric striations are observed in all sections at ×100. *Bar* = 10 μm. (Color figure online)

Histological examinations performed on 0.5 μm thick plastic sections of the MyBP-C DA-1 patient samples are shown in Fig. 2. The morphology of the samples was highlighted by Paragon stain, which renders cellular components dark blue and connective tissue such as collagen pink. At lower magnification the staining of the muscle fibres is seen in blue, and at 100×, the striation pattern due to the sarcomeres in the longitudinal orientation can be clearly recognized in all the samples (both cardiac and skeletal). Light micrographs recorded with 20× objective and 100× oil immersion objective are shown in Fig. 2. The skeletal mutants displayed variable levels of staining for connective tissue (Fig. 2a, b). The amount of connective tissue appears to be more abundant in ss-Y856H (Fig. 2b) compared to ss-W236R (Fig. 2a). The ss-W236R tissue sections also show signs of severe bending of the myofibrils in some regions (indicated by "*" symbol in Fig. 2a). This is not an effect of sample mishandling because the surrounding fibres are normal (marked with a + symbol). This type of misalignment was not obvious for ss-Y856H under the light microscope since most of the fibres in the sample were in the transverse orientation, but was more clearly observed while imaging with EM (Ultrastructure of the diseased tissue).

Histological examination of the cardiac samples MH1, M4 and M9 are shown in Fig. 2c, d and e, respectively. Staining for connective tissue is very clear for MH1 (Fig. 2c), but is less clear for M4 (Fig. 2d) and M9 (Fig. 2e) samples as they were prepared by homogenisation and therefore most of the extra-sarcomeric features are lost. Myofibrillar disarray, a hallmark of the disease, is noticeable for the MH1 sample; this was also evident from hematoxylin and eosin staining of larger cryosections of MH1 (data not shown).

### Ultrastructure of the diseased tissue

**Fig. 3:**
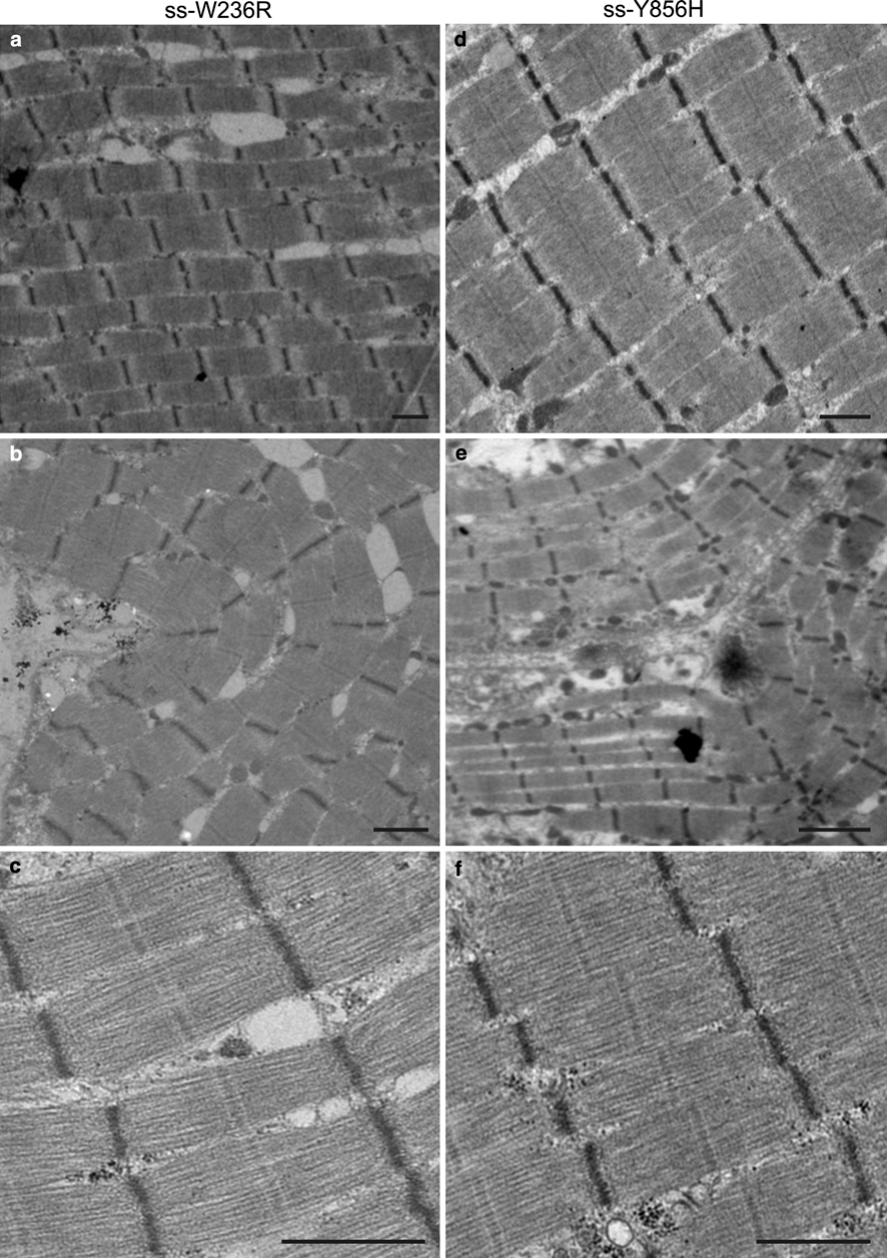
Transmission electron micrographs of the skeletal biopsy samples. Low and medium magnification overview of myofibrillar and sarcomeric ultrastructure of **a**–**c** ss-W236R and **d**–**f** ss-Y856H. **a**, **d** show micrographs of regions with well-aligned and straight myofibrils; **b**, **e** EM of myofibrils showing a “bent” appearance; **c**, **f** EM micrographs of sarcomeres with good visual structure showing well-defined M-bands and Z-discs. *Bar* = 1 μm

**Fig. 4:**
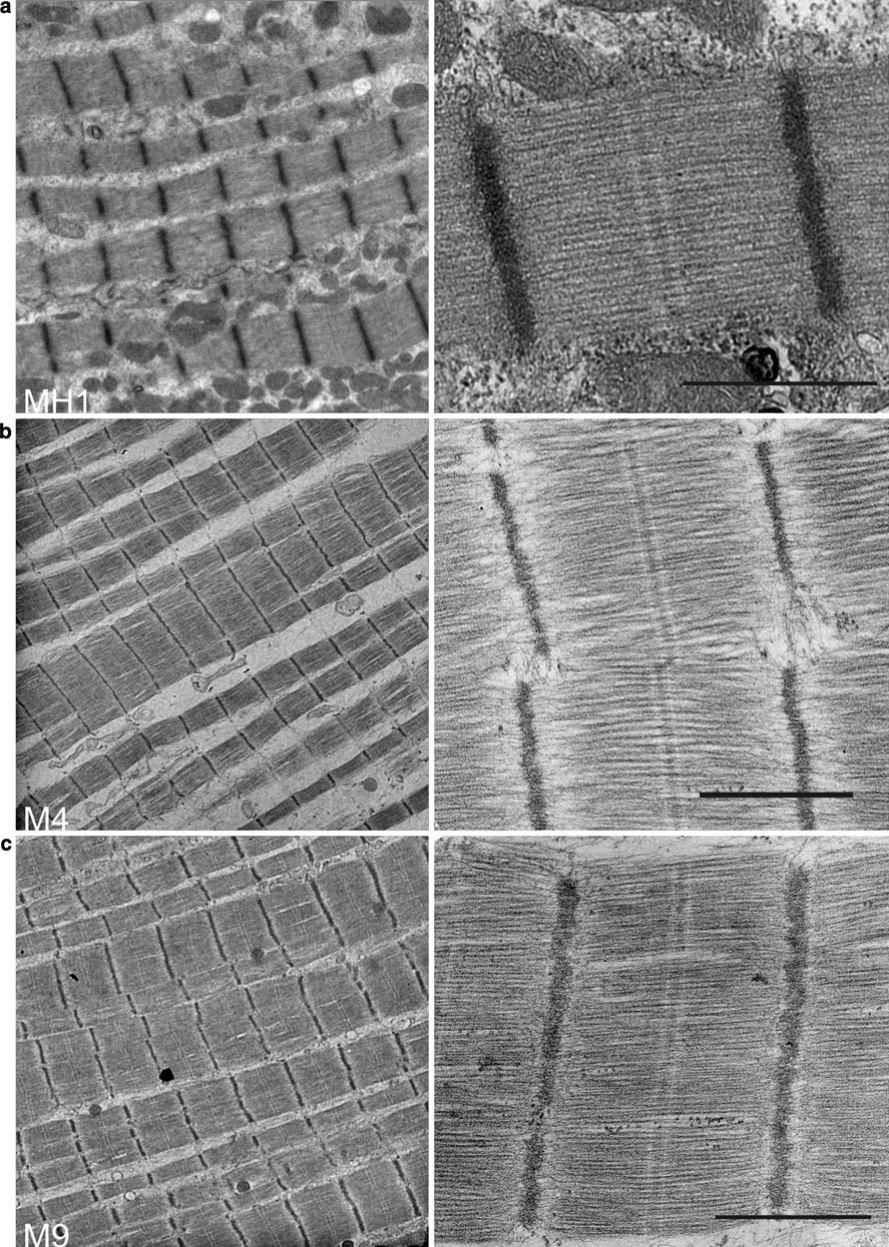
Electron micrographs of cardiac myectomy samples showing low magnification (*left*) and high magnification (*right*) views. **a** (*left*) Low magnification survey of MH1 showing an overview of myofibrillar organisation. **a** (*right*) Micrograph of MH1 showing good, intact sarcomere. **b**, **c** Electron micrographs for M4 and M9 respectively confirm the preservation of sarcomere structure in both samples. Intact sarcomeres with straight M-bands and well-preserved Z-discs can be identified for all three samples. *Bar* = 1 μm

The effects of the MyBP-C gene mutations on the general features and ultrastructural organisation of the diseased skeletal and cardiac muscle was studied by electron microscopy at a range of low-medium magnifications (Figs. 3, 4). Longitudinal sections of the muscle were examined for signs of myofibrillar disarray and disorder within sarcomeres. The electron micrographs of the skeletal mutations (ss-W236R and ss-Y856H) exhibited regions with well-aligned and straight myofibrils (Fig. 3a, d), as well as, myofibrils with a bent appearance (Fig. 3b, e). However, the sarcomere itself is well preserved, and features such as the Z-disc, M-band and I-bands are clearly visible.

Electron micrographs for the cardiac samples MH1, M4 and M9 are shown in Fig. 4a, b, c, respectively. The myofibrillar organisation of these samples appears to be well aligned. There is also no evidence of sarcomeric disarray for all three samples. However the cardiac sarcomeres appear to be contracted, and is especially evident for MH1 and M9. There is also no obvious loss in structure and features such as the Z-disc and M-band, but the I-band region is less clear due to its contracted state (a and c). Contracted sarcomeres are observed despite the addition of 30 mM BDM, a low-affinity myosin inhibitor that selectively inhibits actin-myosin interaction during the sample preparation (Higuchi and Takemori 1989; Ostap 2002) and prevents dissection-induced contracture (Mulieri et al. 1989). Therefore, this shortening of sarcomeres could be a pathological manifestation of the disease causing mutation (Pohlmann et al. 2007).

### Average axial density distribution

**Fig. 5:**
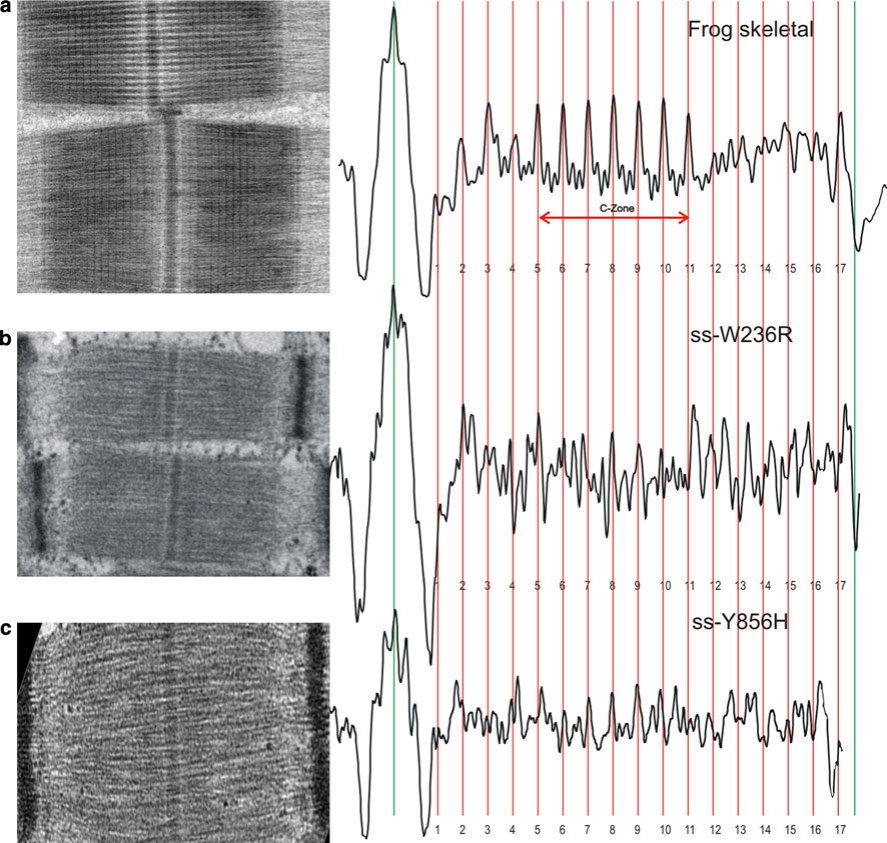
Averaged axial density profile plots of skeletal muscle with *MYBPC1* mutations and control samples. The profile plot from **a** frog sartorius muscle is compared with the mean profile plots of **b** ss-W236R and **c** ss-Y856H. The* left panel* shows an example electron micrograph used to calculate the average profile plots in the* right panel*. The profile plots are aligned at the centre of the M-band and the edge of the A-band (*green lines*) and 17 *red stripes* of 43-nm spacing. **a** Reproduced from Luther et al. (2008) with permission from Elsevier. (Color figure online)

**Fig. 6:**
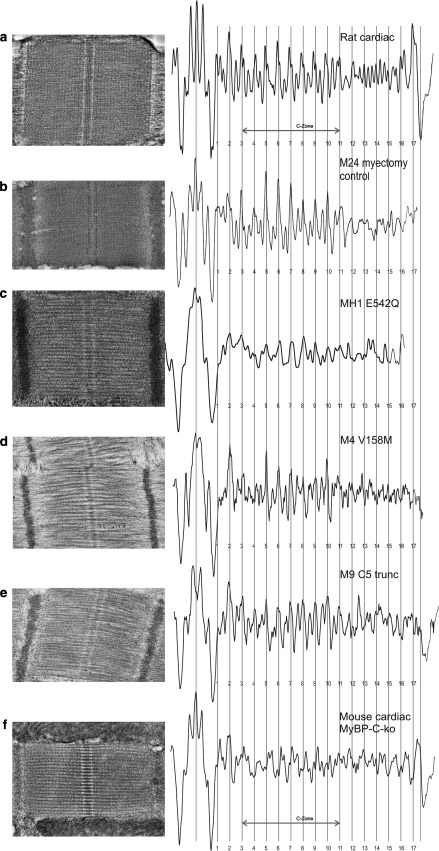
Averaged axial density plot profiles of the cardiac mutants and control samples. The profile plots are precisely lined up as described previously, along the centre of the M-band and the edge of the A-band. The 9 stripes due to cMyBP-C are labelled as the C-zone. **a** Profile plot derived from rat cardiac muscle (papillary) cryosections was compared with the average profile plot for the HCM myectomy samples: **b** M24 myectomy control sample; **c** MH1, **d** M4, **e** M9.* Distinct* and* regular peaks* can be observed in the C-zone of the cardiac mutant samples that coincide with the cMyBP-C axial spacing of 43 nm (*red stripes*). These plots were also compared with **f** mouse cMyBP-C-ko muscle cryosections. MyBP-C stripes are considerably suppressed in the cMyBP-C-ko muscle. **a**, **f** Reproduced from Luther et al. (2008) with permission from Elsevier. (Color figure online)

One-dimensional analysis was carried out on human skeletal and cardiac muscle carrying *MYBPC1* and *MYBPC3* mutations respectively. Axial distribution of MyBP-C in these samples was analysed by averaging profile plots of the A-band over several electron micrographs of half-sarcomere regions by cross-correlation to produce an average axial density profile. The axial density plots for the skeletal and cardiac samples are compared in Figs. 5 and 6, respectively. The averaged half A-band plot was divided into seventeen equally spaced bands of 43 nm intervals, marked by red lines labelled 1–17 in Figs. 5 and 6, although non-myosin protein is known to be present only on stripes 1–11. Luther et al. (2008) have determined previously by immuno-electron microscopy that the C-zone in cardiac muscle is located between stripe 3 and 11, hence the P-zone (proximal) spans stripes 1–3. In these figures, representative electron micrographs of the different samples are shown in the left panel, while in the right panel the plot profiles are arranged to line up at the centre of the M-bands and the edges of the A-band marked by the two green lines. The plots are regular and show peaks of high density in the A-band region. As discussed below, the majority of the peaks within the C-zone coincide with the 43 nm spaced red lines indicating the presence of one layer of myosin crossbridges (crowns) and MyBP-C.

In order to confirm the positioning of these C-zone peaks, the profile plots from the DA-1 skeletal muscle with *MYBPC1* mutations were aligned and compared with the axial density profile plot derived from frog sartorius (fast) skeletal muscle (Fig. 5). Figure 5a shows the profile plot for fast skeletal muscle from frog sartorius (reproduced with permission from Luther et al. 2008). In fast skeletal muscle, the C-zone spans over seven stripes, stripe 5–11, while in slow skeletal muscle, the C-zone spans over nine stripes, stripe 3–11 (Bennett et al. 1986). Since the mutant samples were obtained from abductor hallucis muscle which is composed of type I and type II fibres, we specifically identified type I fibres by measuring the Z-disc width in the electron micrographs. Only sarcomeres in which the Z-discs had widths of 100–140 nm were used in our analysis (Luther 2009). The profile plots for the two mutant samples, ss-W236R and ss-Y856H, show well preserved M-bands (Fig. 5b, c). For both the mutant samples, the presence of MyBP-C is evident from the several 43 nm peaks in the C-zone; however all nine peaks are not equivalent. For sample ss-W236R (Fig. 5b), distinct peaks are present which correlate with positions of the peaks 5–11 in the frog muscle in Fig. 5a, however the amplitude of the peaks is quite variable. Sample ss-Y856H (Fig. 5c) shows stripes at position 5–10 that correlate with the position of the peaks in the frog muscle (Fig. 5a). The irregularities observed in the peak amplitude for ss-W236R could be due to sample preparation artefacts or it could indicate a possible disruption in protein incorporation in those stripes. Further study is required to resolve this.

The cardiac HCM samples (M24, MH1, M4 and M9) are analysed in Fig 6. Their profile plots were aligned with the profile plot derived from rat cardiac muscle (reproduced with permission from Luther et al. 2008). Figure 6a shows the profile plot for the rat cardiac sections, the C-zone spanning 9 stripes from stripe 3–11. The myectomy control sample, M24 (Fig. 6b), shows excellent correlation spatially and in amplitude with the rat cardiac profile and distinct peaks are evident in all the 9 positions. Like the rat cardiac sample, this sample was prepared by cryosectioning as described in Luther et al. (2008); this technique gives particularly high resolution structural detail e.g. it shows the presence of the two layers of myosin crowns between each pair of 43 nm stripe (the 43 nm stripes are a summation of MyBP-C and one layer of myosin crowns). Such detail is often less clear in plastic embedded sections. The mutant samples (Fig. 6c, d, e) also show good spatial correlation with the C-zone peaks but the resolution of detail is variable. The plot for MH1 (Fig. 6c) is regular, with C-zone peaks of similar amplitudes, but only 6 out of the 9 peaks are well matched and the resolution between the peaks is low in this plastic embedded sample. For M4 and M9 (Fig. 6d, e, respectively), the C-zone peaks are clear with better crown detail; for M4 all the C-zone stripes are well matched, except for stripes 4 and 11 which are considerably suppressed. For sample M9 (Fig. 6e), all 9 C-zone stripes are distinct and comparable with Fig. 6a, b.

The axial density plots of the cardiac mutant samples, in comparison with the control skeletal and cardiac models, clearly show evidence of cMyBP-C expression and its incorporation in the C-zone. The sharpness of the peaks further indicates that cMyBP-C is confined to a narrow disc axially and is not altered in the diseased state.

### Comparison with mouse cMyBP-C-ko model

We further compared the cardiac mutant samples with axial density distribution of mouse cMyBP-C-ko muscle (Fig. 6f; reproduced with permission from Luther et al. 2008). Luther et al. looked at the effects of cMyBP-C deficiency on the axial density distribution in the C-zones of the knockout mice. As shown in Fig. 6f, the C-zone peaks at each of the stripes 3–11 are considerably suppressed (Luther et al. 2008). This is in contrast to the distinct, regular peaks observed in the C-zone of the cardiac mutant samples that coincide with the densities corresponding to MyBP-C stripe positions in the rat cardiac and human myectomy control sample.

### Fourier transform analysis of the profile plots

**Fig. 7:**
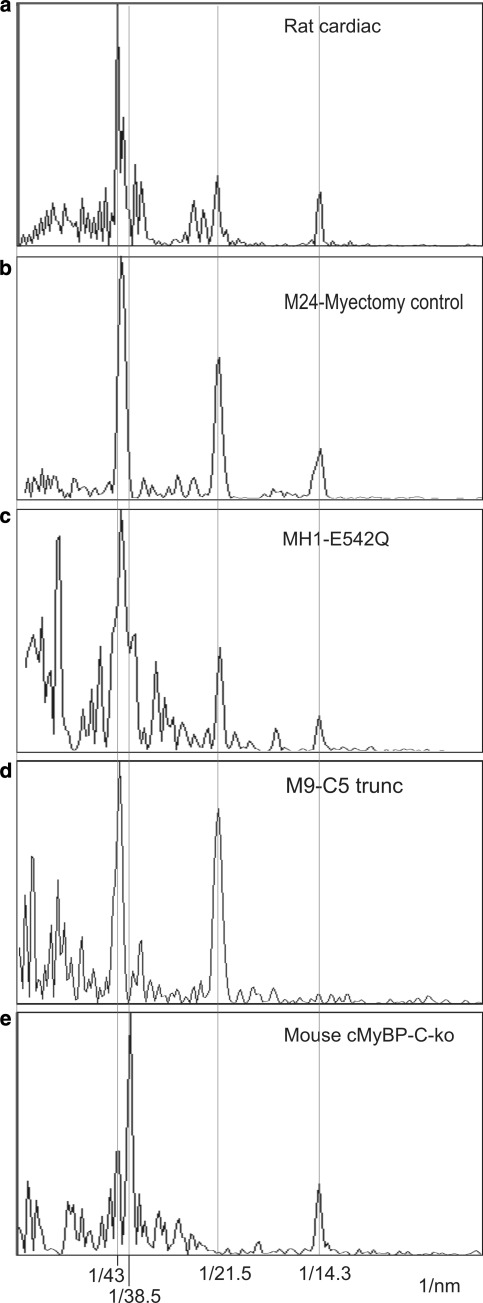
1-D Fourier transforms of cardiac samples **a** Rat cardiac muscle, **b** Myectomy control sample M24, **c** Cardiac HCM samples MH1, **d** M9 and **e** cMyBP-C-ko muscle. The plots are aligned precisely with *red lines* going through the 43, 21.5, 14.3 nm spots (orders of 43 nm) and through the 38.5 nm spot corresponding to troponin. The 43 and 21.5 nm spots are referred to as forbidden meridionals, since they would not be observed if the thick filament symmetry was strictly helical. In MyBP-C-ko cardiac muscle, the 21.5-nm reflection is completely absent and the 43-nm peak is greatly diminished, but they are evident for HCM cardiac samples and rat cardiac muscle. **a**, **e** Reproduced from Luther et al. (2008) with permission from Elsevier. (Color figure online)

One-dimensional Fourier transforms for rat cardiac muscle (reproduced with permission from Luther et al. 2008), cardiac HCM samples M24, MH1 and M9 are shown in Fig. 7a–d, respectively, aligned with the Fourier transform of the mouse cMyBP-C-ko muscle cryosection in Fig. 7e (reproduced with permission from Luther et al. 2008). In the transforms for the HCM samples in Fig. 7b–d, the reflections corresponding to the forbidden meridionals at 43 and 21.5 nm are prominent, and align well with the transform for rat cardiac muscle (Fig. 7a). In contrast, the 1-D transform of the cMyBP-C-ko muscle (Fig. 7e) shows completely absent 21.5-nm reflection and a greatly diminished 43 nm peak. The reflections at 14.3 nm due to the myosin crossbridge repeat are clearly present in all the samples (except for M9). This is a normal reflection on the meridian unlike the meridional 43 and 21.5 nm spots which are “forbidden” spots. Hence the mouse knockout lacking cMyBP-C has very weak forbidden spots, compared to human cardiac muscle with HCM-causing *MYBPC3* mutations. This suggests that although mutant cMyBP-C is not detected in human cardiac samples, the heterozygous allele contributes sufficient cMyBP-C protein to the C-zone banding pattern for this type of structural analysis.

## Discussion

Development of cardiomyopathy and skeletal muscle myopathy due to missing or aberrant MyBP-C protein highlights the importance of studying MyBP-C and understanding its role in the sarcomere. HCM causing *MYBPC3* mutations (both missense and nonsense mutations) are numerous and well documented (Harris et al. 2011). About two-thirds of *MYBPC3* mutations are predicted to generate truncated protein products that have so far not been detected in myocardial samples. Missense mutations resulting in single amino acid substitutions have been identified to occur throughout the protein sequence. Cardiac MyBP-C with the E542Q missense mutation (MH1 in this study) showed evidence of sarcomeric incorporation comparable to endogenous cMyBP-C when expressed in rat fetal cardiomyocytes (Flavigny et al. 1999). However there is still no direct experimental evidence from patient biopsy samples supporting a poison peptide mechanism for missense *MYBPC3* mutations. In fact levels of cMyBP-C measured from myectomy samples appear to be reduced in HCM samples compared to the controls, supporting haploinsufficiency as the disease-causing mechanism (Marston et al. 2009, 2011; Van Dijk et al. 2009).

To date only two slow skeletal muscle *MYBPC1* mutations have been identified; these are missense mutations and both occur in highly conserved regions. The W236R mutation occurs in the MyBP-C motif, while the Y856H mutation is located in a partially exposed region of the C8 domain (Gurnett et al. 2010). Unlike cMyBP-C in HCM patients, the level of total ssMyBP-C was not found to differ between patients with the W236R and Y856H *MYBPC1* mutations and controls. Also preliminary studies carried out using GFP-tagged ssMyBPC showed normal localisation of wild type ssMyBP-C as well as mutant ss-W236R and ss-Y856H in mouse epitrochlearis muscle; in contrast there was poor localisation of MyBP-C containing homologous cardiac mutations (Gurnett et al. 2010).

The results discussed here provide useful insights into the structural consequence of MyBP-C gene mutation. The following observations emerge for both the cardiac and skeletal muscle specific mutation:

1. The ultrastructure of the sarcomere in longitudinal sections appears to be well preserved despite the phenotype of muscle disorder.
2. Image analysis confirms MyBP-C incorporation into the A-band and sharpness of peaks further indicates that it is confined to a narrow disc axially.
3. Comparison with the cardiac MyBP-C ko model provides no indication of altered axial distribution in the HCM samples.

The axial distribution that we observe is due to projection of density through the depth of the section. If all MyBP-C molecules lie in the same plane in longitudinal view, it would give rise to sharp peaks in averaged plot profiles. If there is movement in MyBP-C N-terminal “arms” within the same plane this would not affect the axial density and the stripes would remain sharp. Similarly, if a few N-terminus domains move out of the plane, this would reduce the density of the peaks but only by a little and still show clear peaks by our averaging method. A stripe would not be visualised only if the MyBP-C arms move out of the plane at the C-terminus of the protein. Our results suggest that MyBP-C remains confined to a disc and structurally intact at the C-terminus. But the state of N-terminus cannot be commented upon from these results and electron tomography of transverse sections of the muscle is required to investigate the disposition of N-terminal half of MyBP-C.

The 1-D Fourier transforms of the cardiac profile plots and comparison with cardiac MyBP-C ko model further confirms the notion that cMyBP-C is incorporated and axially distributed correctly. X-ray diffraction patterns of striated muscle show forbidden meridional reflections corresponding to the first, second, fourth, etc. orders of 42.9 nm; the presence of which may be caused by variations in the myosin head spacing or the presence of accessory proteins such as MyBP-C at every third myosin head repeat or a combination of both. For the cardiac HCM samples both the 21.5 and 43 nm peaks occur prominently, which are significantly suppressed in the cMyBP-C ko muscle. Thus the forbidden meridionals appear to be unaffected in these HCM causing cardiac mutations, suggesting the thick filament arrangement is not disrupted.

However, these data do not explain the apparent reduction (~20 %) in the amount of cMyBP-C in HCM patients with *MYBPC3* mutations (Marston et al. 2009). With the heterozygous allele contributing a high level of wild type cMyBP-C, such a reduction (~20 %) may not be resolved by the methods used in this study. The samples used for electron microscopy with the current methods have a lot of variability and the absolute levels of density are hard to determine unambiguously. In addition, artefacts from the muscle preparation techniques increase the noise in the density profiles. Samples prepared by cryosectioning (Luther et al. 2008) or fast freezing and freeze substitution (Luther et al. 2011) have superior preservation and can even show the 14.3 sub-bands arising due to the crown densities in each 43 nm period. However this is difficult to achieve for human biopsy/myectomy samples. Plastic sections prepared by conventional processing methods show good morphology but fine details like the 14.3 nm sub-bands are harder to preserve. Hence in this study the banding pattern reliably demonstrates the location of proteins rather than their relative abundance. To assess the amount of protein from the density in the electron microscope, more specialised methods are required like mass measurement by scanning transmission electron microscopy (e.g. Liversage et al. 2001).

In summary, we describe here the first EM based analysis of human cardiac and skeletal muscle samples with MyBP-C gene mutations. From our current method of analysis, incorporation and axial density distribution of the protein appears to be unaffected, as is evident from the axial density plot and the 1-D Fourier transform.

## Abbreviations

MyBP-C: Myosin binding protein-C
HCM: Hypertrophic cardiomyopathy
DA-1: Distal arthrogryposis type 1
EM: Electron microscopy
IgI: Immunoglobulin
FnIII: Fibronectin type III
LMM: Light meromyosin portion of the myosin rod
cMyBP-C: Human cardiac myosin binding protein-C
ssMyBP-C: Human slow skeletal myosin binding protein-C
wt: Wild type
ko: Knock out
